# Paramedics Performed Sonographic Identification of the Conic Ligament—A Prospective Controlled Trial

**DOI:** 10.3390/diagnostics15101296

**Published:** 2025-05-21

**Authors:** Johannes Weimer, Christopher David Chrissostomou, Christopher Jonck, Andreas Michael Weimer, Carlotta Ille, Lukas Müller, Liv Annebritt Lorenz, Marie Stäuber, Thomas Vieth, Holger Buggenhagen, Julia Weinmann-Menke, Maximilian Rink, Julian Künzel

**Affiliations:** 1 Department of Internal Medicine I, University Medical Center of the Johannes Gutenberg University Mainz, 55131 Mainz, Germany; julia.weinmann-menke@unimedizin-mainz.de; 2 Rudolf Frey Learning Clinic, University Medical Center of the Johannes Gutenberg University Mainz, 55131 Mainz, Germany; cjonck@students.uni-mainz.de (C.J.); carlotta.ille@outlook.com (C.I.); mstaeube@students.uni-mainz.de (M.S.); thovieth@uni-mainz.de (T.V.); buggenha@uni-mainz.de (H.B.); 3 Department of Otorhinolaryngology, Head and Neck Surgery, University Hospital Regensburg, 93053 Regensburg, Germanymaximilian.rink@klinik.uni-regensburg.de (M.R.); julian.kuenzel@klinik.uni-regensburg.de (J.K.); 4 Clinic for Trauma and Reconstructive Surgery, University Clinic Heidelberg, 69118 Heidelberg, Germany; andreas.weimer@kkh-bergstrasse.de; 5 Department of Diagnostic and Interventional Radiology, University Medical Centre of the Johannes Gutenberg University Mainz, 55131 Mainz, Germany; lukas.mueller@unimedizin-mainz.de; 6 Department of Radiation Oncology and Radiotherapy, University Medical Center of the Johannes Gutenberg University Mainz, 55131 Mainz, Germany; liv-annebritt.lorenz@unimedizin-mainz.de

**Keywords:** paramedics, airway management, ultrasound training, cricothyrotomy, ligamentum conicum, conic ligament, education, ultrasound-guided interventions, direct observation of procedural skills

## Abstract

**Background/Objectives**: Acute obstructions of the upper respiratory tract are emergencies that may require a cricothyrotomy as ultima ratio. For this, precise identification of the conic ligament is essential. Point-of-care ultrasound (POCUS) offers a reliable tool for anatomical localization in challenging cases and could be used by a range of emergency medicine workers. This prospective, controlled observational study assesses the development of competencies of paramedics (PMs) in point-of-care ultrasound (POCUS) assisted identification of the conic ligament after structured training, and compares their competence level to emergency physicians (EPs). **Methods**: PMs and a control group of EPs received an identical structured training program as part of an ultrasound course. It included a 10-min theoretical introduction, a 10-min video, and a 45-min practical session with ultrasound devices. Questionnaires and a practical test assessed both group’s previous experiences, satisfaction with training, and the development of subjective and objective competencies before (T_1_) and after (T_2_) the training. **Results**: A total of 120 participants (N = 92 PMs and N = 28 EPs) participated. A minority had previously performed a cricothyrotomy even in training settings (PMs 17%; EPs 11%), and none had identified the conic ligament using POCUS. The study group’s subjective and objective competencies increased significantly (*p* < 0.001). At T2, the study group demonstrated comparable subjective (*p* = 0.22) and objective (*p* = 0.81) competencies to those of the control group. The study group needed significantly (*p* < 0.01) less time to perform the DOPS. While both groups were satisfied with the study material (PMs 2.2 ± 1.2 vs. Eps 1.6 ± 1.0; *p* = 0.02) and the training (PMs 1.8 ± 1.0 vs. EPs 1.4 ± 0.7, *p* = 0.03), the study group rated both significantly better. **Conclusions**: After structured training, paramedics successfully identified the conic ligament using POCUS comparably to emergency physicians. Integrating POCUS into paramedic training may improve prehospital airway management and enhance patient safety. Further studies should investigate long-term skill retention and real-life application.

## 1. Introduction

### 1.1. Background

Emergency medicine faces special challenges in preclinical airway management [1], especially in so-called “difficult airway” situations [2,3]. Acute obstructions of the upper respiratory tract by foreign bodies, trauma, tumors, or severe infections require quick and precise handling. During a “Cannot-Intubate-Cannot-Ventilate” (CICV) situation, in which neither intubation nor ventilation is possible, the emergency cricothyrotomy remains as a last resort to secure the airways [2,3,4,5,6]. While a viable option for airway management in a hospital setting, an emergency tracheotomy is generally unrealistic in prehospital care due to the increased risk of bleeding and the typically limited experience of practitioners in this setting [7]. For this reason, it is essential that preclinical emergency physicians (EPs) and the assisting paramedics (PMs) have the necessary amount of theoretical and practical knowledge to perform a cricothyrotomy.

During a cricothyrotomy, the trachea is accessed by puncturing the conic ligament between the thyroid and cricoid cartilage [5,6,8,9]. The conventional identification of this ligament is done by palpation [10]. However, this method is prone to errors, especially in patients with aberrant or complex neck anatomy, such as with obesity or swelling [11]. Sonography is a promising alternative to landmark identification by palpation and is included in current guidelines for airway management [2,12,13,14,15,16,17,18]. Previous studies have shown ultrasound assisted identification of the conic ligament to be significantly more precise than palpation. Especially in the case of complex anatomical conditions, sonography can significantly improve the accuracy of landmark identification [13,14,15,16,17,18].

Cricothyrotomies are rare emergency medical procedures, with reported incidences ranging from 0.5% to 1% of all emergency airway interventions in prehospital and trauma care settings [18,19] that preclude structured training on the job, emphasizing the need for alternative training programs. Still, the training of sonographic identification of the conic ligament has only been evaluated in a few studies until now, either in the form of a structured training concept in head and neck sonography [20] or as a short theoretical-practical introduction [21,22,23,24,25]. In general, cricothyrotomies are usually practiced on models (simulators or cadavers) for only a few repetitions, leading to a steep learning curve to implement in real-world care [26,27,28]. Recently, virtual reality approaches are also being used to teach the relevant skills [29], though the training of PMs focuses on the identification of anatomical landmarks by palpation on healthy human subjects. Sonography has not been integrated into this training [30].

### 1.2. Research Problem and Aim

The development of portable ultrasound devices [31,32] opens up new opportunities for their use in preclinical emergency medicine, for example in the evaluation of difficult airways [2,33,34,35]. While the use of emergency sonography is routine for EPs today, PMs could also apply some ultrasound techniques [36,37]. The integration of sonography into PMs’ basic and continued professional training could support EPs, improve decision-making processes, and support the differential diagnosis skills of PMs. This is especially crucial if no EP is available [38]. Despite these benefits, sonography has only been used by PMs to a limited extent due to its minimal impact on therapeutic decision making for PMs and the absence of structured, uniform training concepts [39]. Previous studies have found the application of sonography by PMs to be practicable, but there is no evidence for the actual feasibility and effectiveness in specific emergency situations like the one investigated in this study [36,37,40,41,42,43,44,45].

Current approaches to the implementation of sonography into the workflow of PMs include the training of simple, standardized techniques, such as thoracic sonography to assess dyspnoea [43]. Rare, but critical emergencies are thus far unstudied applications in which specific and easy-to-learn sonographic techniques could offer significant advantages. One example is the sonographic identification of the conic ligament for a cricothyrotomy. In life-threatening situations, first responders being able to identify the cricothyroid ligament represents a valuable skill and significantly improves the chances of a successful cricothyrotomy.

The present study aims to assess the effectiveness of paramedic training in sonographic identification of the conic ligament. The subjective and objective increase in competency are evaluated after a structured training program. The competencies of PMs after this training are compared to those of EPs who undertook the same training. In addition, the quality of and satisfaction with the training program and the study materials are assessed by the participants. The main hypothesis of the study is that after completing the training program, PMs will be able to identify the conic ligament using sonography just as reliably and precisely as EPs.

## 2. Material and Methodology

### 2.1. Study Design, Participant Recruitment, and Inclusion Criteria

**Figure 1:**
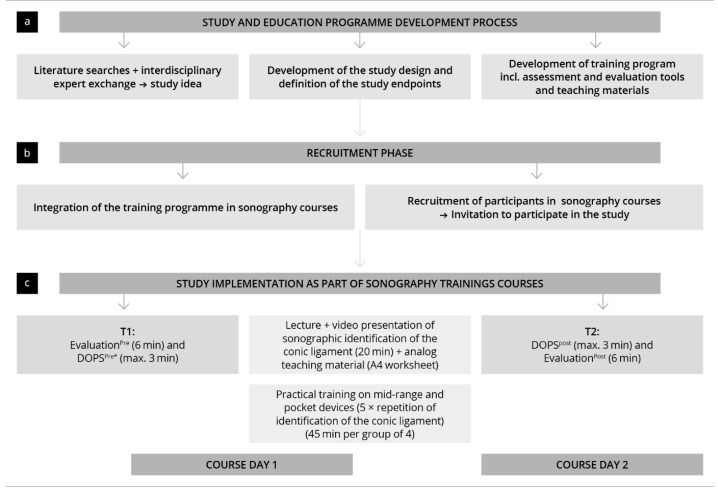
Illustration of the study process, including recruitment, materials, methodology, and measurement timeline. * This test was not performed by the control group.

This prospective, controlled observational study was planned and carried out from 2023 to 2024 [46]. The development of subjective and objective skills in the sonographic detection of the conic ligament were investigated as part of a sonography training course for German PMs [45]. The participants completed an additional training program to the typical course. The development of competencies was evaluated at two time points: before the start of the training (T_1_ = pre-training) and after completing (T_2_ = post-training). Both subjective assessments were recorded using questionnaires (Evaluation_pre_ and Evaluation_post_). Objective skills were graded by a direct observation of procedural skills (DOPS) test (DOPS_pre_ and DOPS_post_) [47]. The evaluation and testing instruments used were developed through the consensus of sonography experts and didactics based on previous work and current recommendations [47,48]. The subjective competency development (T_1_ vs. T_2_) and the level of competency achieved (T_2_) in the study group was compared to a control group of EPs who also completed the training during an ultrasound course [49]. The study protocol and the assessment times are shown in Figure 1.

The participants were recruited between 2023 and 2024. Completion of all tests and evaluations as well as informed consent to participate in this study were inclusion criteria. Non-consent for participating in the study had no influence on participation in the training.

The primary endpoints of the study were the objective increase in competence between T_1_ and T_2_, as well as the level of competency of the participants in the sonographic detection of the cricothyroid ligament at time T_2_ compared to the control group as measured by DOPS. Secondary endpoints included the subjective increase in competency (evaluated with a questionnaire with 7-level Likert answering formats), the demonstration of laryngeal landmarks and the sonographic guided identification of the conic ligament, and a general assessment of the training concept.

The study was reported to the Regensburg ethics commission, which issued a declaration of dispensability.

### 2.2. Training and Study-Materials

As part of a two-day ultrasound course [45,49], participants underwent a specially developed training program for sonographic detection of the ligamentum conicum [20]. This program consisted of several didactic and practical components:

- 10-min theoretical training (lecture);
- A worksheet and complementary 10-min video presentation of sonographic instructions;
- 45-min practical training in groups of 4.

**Figure 2:**
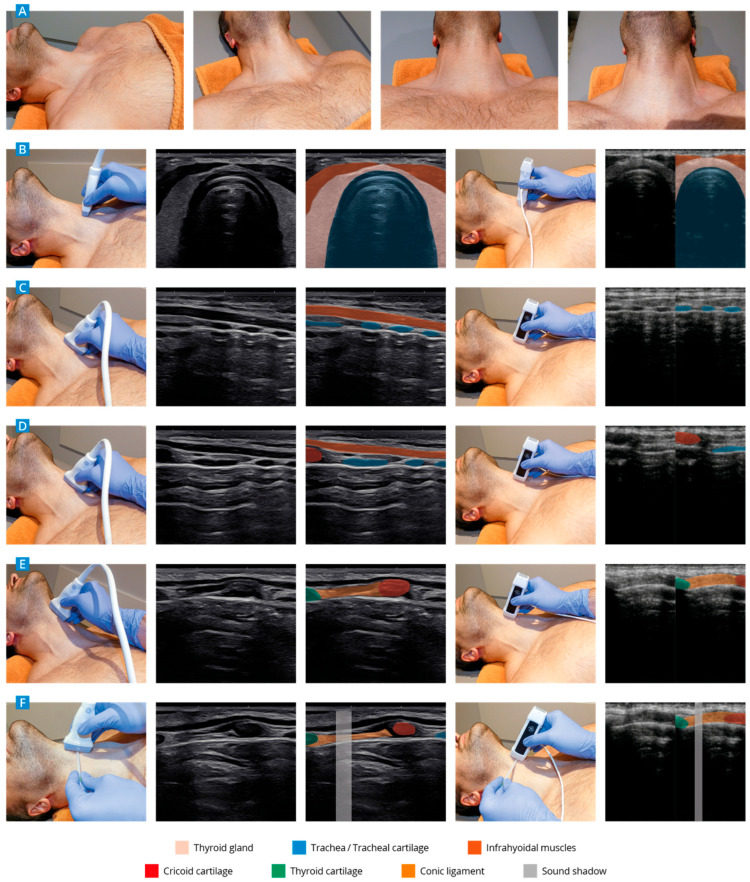
The technique for sonographic identification and indication of the conic ligament with a medium class device is depicted on the left and a pocket device on the right side (based on [14,15]). After positioning (reclination) of the patient (**A**), the trachea is visuallized in transverse section at the level of the thyroid (**B**). The probe is turned 90 degrees clockwise for the representation of the tracheal cartilage (**C**). The probe is moved cranially for visualisation of the cricoid cartilage (**D**), the thyroid cartilage (**D**), and the identification of the conic ligament (**E**). The use of a cannula to mark the conic ligament (acoustic shadow) is depicted (**F**).

The 10-min theoretical session included a structured presentation covering relevant neck anatomy (thyroid and cricoid cartilage, trachea, and conic ligament), the indication and urgency of cricothyrotomy, limitations of landmark palpation, and the role of sonography in airway management. The session also introduced the “string-of-pearls” sonographic technique for identifying the airway structures [14]. The 10-min instructional video visually reinforced the theoretical content and demonstrated the standardized ultrasound procedure. It included probe positioning, image optimization, anatomical orientation, and the identification and marking of the conic ligament on a healthy volunteer. The practical training included the application of the “string of pearls” technique [14] for sonographic identification of the conic ligament, as described in the worksheet (see Figure 2). The participants worked with two ultrasound devices of different categories: a medium-class device (GE Logiq e, General Electric Company, Boston, MA, USA) and a pocket ultrasound device (VSCAN Extend; General Electric Company, Boston, MA, USA) [50]. Under the guidance of experienced ultrasound instructors, the conic ligament was visualized sonographically and the spot for possible access was indicated with a pointer. No puncture was carried out on the subjects as part of the course. Every participant went through five training rounds.

The practical training was supervised by ultrasound tutors, including both physicians as well as experienced paramedics. All tutors had formal qualifications in emergency and head and neck ultrasound, as well as substantial teaching experience in accredited sonography courses. To ensure standardization across all training sessions, all instructors were provided with a uniform curriculum, including a detailed script, standardized lecture slides, a 10-min instructional video, and written procedural checklists. Instructors received a joint briefing prior to the course to align teaching approaches. This guaranteed a consistent learning experience for all participants, independent of instructor or session.

### 2.3. Questionnaires and Practical Tests

#### 2.3.1. Questionnaires

The pre- and post-evaluation questionnaires covered various topics using multiple items (see Supplementary Materials S1). The topics were “personal data”, “previous experience”, “subjective competency assessment”, “evaluation of training”, and “evaluation of study materials”. Responses were recorded with 7-level Likert answering formats (1 = “very good”/“completely agree” to 7 = “very poor”/“completely disagree”). Dichotomous questions (“yes”/“no”) and free-text fields were also used. Data were collected in written form.

#### 2.3.2. Practical Test

To measure practical skills in sonographic detection of the conic ligament, a direct observation of procedural skills (DOPS) test (38 items) was developed based on previous studies [20,48] and the technique of Kristensen et al. [51] (see Supplementary Materials S2). The competency aspects included in the DOPS are “positioning and landmarks” (max. 4 points), “transducer handling and image optimization” (8 points), “identification and labelling of sonographic landmarks” (8 points), “examination procedure” (12 points), and “sonographic guided location of the conic ligament” (6 points). A total DOPS score was calculated as a percentage of the maximum achievable score. In addition, the total time required for completion was recorded. The time limit for completing the DOPS test was 3 min, after which time the test was aborted.

The study group completed the DOPS test both before training (day 1) and one day after training (day 2). The tests were performed on one of the three different human volunteers without pathological findings in the examination area using a pocket ultrasound device (VSCAN Extend; General Electric Company, Boston, MA, USA). These devices enable rapid and flexible diagnostics, particularly in emergency situations [14,31,50]. The DOPS were evaluated by didactically trained ultrasound tutors as scribed above. For validation purposes, the DOPS were administered by a primary examiner and a secondary examiner. Both examiners were blinded to each other.

#### 2.3.3. Statistics

The written evaluation and examination forms were stored in spreadsheets (Microsoft Excel). All statistical analyses and diagrams were produced with RStudio (RStudio Team [2024], RStudio: Integrated Development for R, RStudio, PBC, http://www.rstudio.com, last accessed on 29 October 2024) using R 4.0.3 (A Language and Environment for Statistical Computing, R Foundation for Statistical Computing, http://www.R-project.org, last accessed on 29 October 2024). Binary and categorical output parameters were presented as absolute numbers and percentages (relative to the maximum value) or in percentage ranges. Continuous data were expressed as median and interquartile range (IQR) or as mean (MW) and standard deviation (SD). In addition, delta values (Δ) were calculated for the change between T1 and T2. Categorical parameters were compared using Fisher’s exact test. The Mann–Whitney test was used for continuous parameters. A *t*-test was used to analyze the influence of potential factors on DOPS scores and DOPS time (e.g., gender, educational level, preclinical experience, attended ultrasound diagnostic courses, number of ultrasound examinations without supervision, cricothyrotomies observed, sonographically assisted cricothyrotomies observed, cricothyrotomies performed without supervision, and previous experience with pocket devices). *p*-values < 0.05 were considered statistically significant. In addition to significance testing, Cohen’s d effect sizes were calculated for the main subjective and objective outcome measures (e.g., total DOPS scores, time and self-assessed competency) to quantify the magnitude of observed differences and support interpretation of clinical relevance. To validate the DOPS test, the Spearman rank correlation coefficient was calculated to determine interrater reliability, which was used as a measure of the agreement between the ratings of the primary and secondary examiners.

## 3. Results

### 3.1. Demographics and Previous Experience

The data of 120 participants were analyzed (N = 92 PMs as study group and N = 28 EPs as control group). Demographics and previous training are summarized in Supplementary Materials S3. Both groups did not differ in average age (study group 35 ± 10 years vs. control group 37 ± 12 years, *p* = 0.66), although the proportion of male participants was higher in the study group (study group 79% vs. control group 43%, *p* < 0.01). Both the proportion of participants who worked in a prehospital setting (*p* < 0.01) and the number of handled preclinical cases in the past (*p* < 0.01) were significantly higher in the study group. In contrast, significantly more participants in the control group attended ultrasound courses previously (study group 10% vs. control group 75%, *p* < 0.01). In addition, the average number of ultrasound examinations performed in general (study group 5 ± 14 vs. control group 554 ± 945; *p* < 0.01) as well as the number of sonographic examinations of the larynx/tracheal region (study group 0.2 ± 0.9 vs. control group 19 ± 52; *p* < 0.01) were significantly higher in the control group. Participants in the control group performed more tracheotomies without supervision (study group 4% vs. control group 43%, *p* < 0.01), while cricothyroidotomies (including during training sessions on simulators) were rare in both groups (study group 17% vs. control group 11%, *p* = 0.53). No participant had previously performed a sonographically assisted cricothyroidotomy without supervision. Approximately half of both groups had previous experience with pocket devices (*p* = 0.14), although the number of examinations performed with pocket devices was significantly higher in the control group (*p* = 0.01).

### 3.2. Practical Skills Development

**Figure 3:**
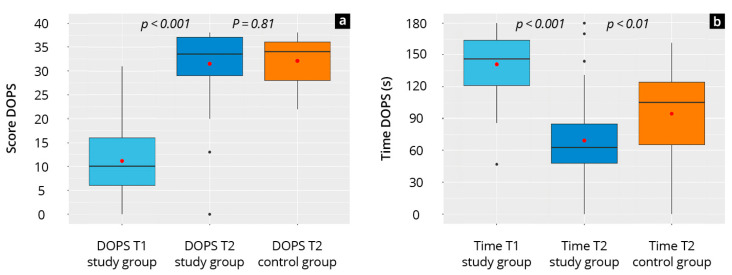
Boxplots of the objective data required for the study and control group at time points T_1_ and T_2_. (**a**) total DOPS scores; (**b**) time required. The red dot shows the mean. For better interpretation of variability, 95% confidence intervals (CI) are reported as follows: study group DOPS score—T1 CI [1.0–29.5], T2 CI [21.0–38.0]; study group time—T1 CI [96.0–223.2], T2 CI [33.3–140.8]; control group DOPS score (T2)—CI [22.6–38.0]; control group time (T2)—CI [49.9–159.9].

Figure 3 and Supplementary Materials S4 show the DOPS results and the time required for completion. The study group showed a significant increase in competencies in all tested areas from T_1_ to T_2_ (*p* < 0.001, d = 2.63). This increase was greatest in the competencies “sonographic guided location of the conic ligament” (Δ 68%) and “structure recognition” (Δ 69%). Except for “transducer handling + image optimization” (study group 86% vs. control group 90%; *p* = 0.02), the competencies at T_2_ were approximately the same in both groups. Furthermore, the study group required significantly less time to complete the DOPS at T_2_ than at T_1_ (T_1_: 158 ± 37 s, T_2_: 70 ± 34 s, *p* < 0.001, d = 2.48) and less than the control group at T_2_ (T_2_: 94 ± 43 s, *p* < 0.01).

### 3.3. Validation of Practical Test

A total of 49 DOPS assessments were independently rated by two trained raters. The interrater reliability of the total score of the DOPS between the assessment of the main examiner and that of the secondary examiner was high (Spearman’s rank correlation coefficient ρ = 0.93).

### 3.4. Factors Influencing DOPS Results and Time to Completion

In the study group, the participant working in a preclinical setting was a significant influencing factor (*p* = 0.009) for a higher test score in the DOPS_T1_. No other significant effects were found for the remaining factors. A complete list of all tested variables and *p*-values is available in Supplementary Materials S5 (study group) and S6 (control group).

### 3.5. Subjective Development of Competency

**Figure 4:**
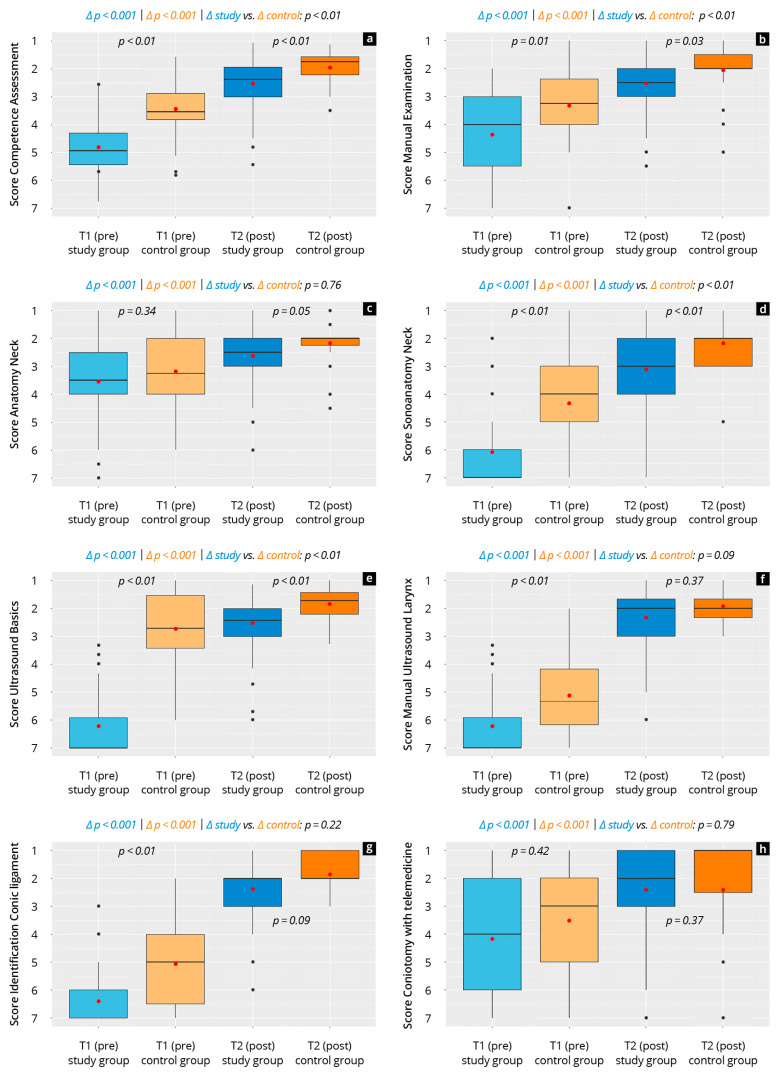
Subjective development of competencies of the study and control group at time points T_1_ and T_2_. The red dot shows the mean. Each diagram of four boxplots shows the results in one competency: (**a**) competency assessment, (**b**) manual examination, (**c**) anatomy of the neck, (**d**) sonoanatomy of the neck, (**e**) ultrasound basics, (**f**) manual ultrasound of the larynx, (**g**) identification of conic ligament, and (**h**) Cricothyrotomy with telemedicine.

Figure 4 and Supplementary Materials S7 show the results of the participants’ subjective competency development. The study group rated their competencies at T_1_ and T_2_ as significantly worse (*p* < 0.01) than the control group in all areas except for anatomy and in the overall score. In both groups, a significant (*p* < 0.001) increase was observed in all competencies from T_1_ to T_2_ (study: d = 2.56, control: 1.69). The study group’s competency gains in the overall score and in all main scores except for anatomy and laryngeal sonography was significantly (*p* < 0.01) higher than in the control group. In both groups, the greatest increase was measured in the competency “laryngeal sonography”.

### 3.6. Evaluation of Training and Study Materials

The study material (study group 2.2 ± 1.2 vs. control group 1.6 ± 1.0, *p* = 0.02) and the training (study group 1.8 ± 1.0 vs. control group 1.4 ± 0.7, *p* = 0.03) were rated positively by both groups, but significantly better in the study group.

## 4. Discussion

### 4.1. Objective and Subjective Competencies

Various previous studies have examined training options for partial aspects of a cricothyrotomy and emphasized the importance of a standardized approach and effective communication between involved professionals. In this study, significant increases in subjective and objective competencies were observed in the training scenario after just a few repetitions of the training [20,26,27,30]. The findings of previous studies in which intensive care personnel performed a total of five cricothyrotomy simulations on a training dummy showed improvement in their performance with each attempt [26]. This finding was replicated in this study. The study group showed significant improvement from T_1_ to T_2_ in all tested objective and subjective competencies and a comparable and high level of competency to the EPs control group after training. The increase in competency in our study was particularly high for “sonographic guided identification of the conic ligament”, implying that POCUS-guided landmark identification can be learned by PMs as a part of ultrasound courses.

The study group required less time to complete the DOPS test than the control group. This could be due to the training effect and to the fact that PMs use case simulations in preclinical training and may therefore internalize decision-making processes faster. However, the average DOPS duration of the study group at T_2_ (94 s) would still be too long for an acute emergency. To ensure effective airway management, a complete cricothyrotomy should ideally be completed within 60 s [2,26]. It is important to consider that the DOPS test in the training setting presented here included additional didactic elements such as image optimization, precise adjustment of standard incisions, and the naming of anatomical landmarks. In a real emergency setting, trained personnel would likely skip these non-essential steps, focusing solely on rapid identification and marking of the ligament. Therefore, it is reasonable to assume that the procedure time could be further reduced under real-life conditions. Nonetheless, this underscores the importance of ongoing training to internalize the necessary steps and improve response speed under pressure. Aside from its applicability in the acute setting, previous studies have already shown that improved sonographic skills in identifying the conic ligament also led to improved palpation skills [12]. This positive general training effect is another advantage of the structured training of PMs in this method.

Still, the handling of ultrasound devices requires in-depth basic training. The EP control group performed better in “transducer handling and image optimization”, likely due to their greater prior experience with sonography and the use of pocket devices. This highlights the need for the integration of POCUS into continuing education programs for PMs [36,40,43,44,45].

Subjective competencies increased significantly in both groups, with the increase being higher in the study group. This could be due to PMs having lower self-efficacy expectations regarding ultrasound application at the beginning of the training or which may be explained by the Dunning–Kruger effect: individuals with limited experience who are aware of their knowledge gaps tend to underestimate their abilities [52]. The structured training and practical application may have significantly increased participants’ confidence in their competence. This change aligns with Bandura’s theory of self-efficacy. In particular, the practical experiences (mastery experiences) and the positive feedback from the instructors (verbal encouragement) may also have played a role in increasing the subjective feeling of competency [53]. The large subjective increase in “laryngeal sonography” competency recorded in both groups was remarkable. This indicates that both groups had received little training in this area to date and that the topic offers a considerable potential for improvement gains in clinical practice [13,14,15,16,17,18]. The realistic self-assessment of competency could have been favored by high learning motivation, as has already been shown in previous studies. A realistic assessment of one’s own abilities is closely linked to increased intrinsic motivation [54]. Nevertheless, a structured POCUS curriculum for emergency medical personnel significantly improved not only practical skills but also confidence in competence, as shown before [45]. These findings support the assumption that the subjectively perceived increase in competence in the present study may also have been influenced by the participants’ high learning motivation.

A central question in prehospital emergency medicine concerns is in which areas PMs can achieve competencies comparable to those of EPs through targeted training. Prior research has indicated that PMs can achieve competencies comparable to those of EPs in certain areas of emergency medicine following structured training programs, for instance in use of analgesia [55]. Still, highly invasive procedures should be led by EPs [56]. The present study extends this important comparison to sonographic landmark identification for cricothyrotomy, thus expanding the evidence for the successful use of POCUS by PMs. Previous studies, also have demonstrated that non-physician providers can acquire basic POCUS skills through structured training programs [36,43]. Our findings are consistent with these results and extend them by showing that paramedics can also be trained to perform a focused, anatomically complex task—identifying the conic ligament for potential emergency cricothyrotomy—within a brief, standardized format. Unlike most prior studies, which focused on diagnostics or general image acquisition, our study addresses a high-stakes, procedure-oriented application. This represents a novel contribution to the field and supports the feasibility of targeted POCUS training even in time-limited educational settings. This marks an important step towards a broader implementation of POCUS in the emergency services. In real-life emergency scenarios, the execution of a cricothyrotomy is often complicated by a range of anatomical and procedural challenges. Known complications include airway hematoma, misplacement of the airway device, and delayed obstruction due to tissue swelling or bleeding at the incision site [57]. Additionally, ultrasound-based identification of the cricothyroid membrane can be significantly impaired in patients with difficult anatomy—such as those with obesity, subcutaneous emphysema, neck trauma, or previous surgery—due to limited visualization or altered landmarks [13].

### 4.2. Training

POCUS is increasingly recognized as an important tool in emergency medical services, increasing the need for structured training on the use of POCUS in prehospital settings [36,37,40,41,42,43,44,45]. The training concept in this study was positively evaluated by both groups and proved effective. A particular aspect worth highlighting is the successful mix of theoretical and practical components [58]. The combination of live lectures, videos, and supervised practical exercises created an effective learning environment, a feature that was also emphasized in other successful POCUS training concepts [20,45]. The training concept is transferable to other user groups and could be integrated into existing training and continuing education programs for emergency medical personnel.

### 4.3. Outlook

Airway management is time-critical; hence, a cricothyrotomy could be indicated in extreme situations before or during the arrival of EPs. Cricothyrotomy sets should be immediately available on all ambulances and personnel should be appropriately trained. POCUS can assist in the emergency application of cricothyrotomies. The implementation of POCUS in prehospital settings faces barriers such as device costs, training logistics, and time constraints in emergencies.

To improve PMs’ POCUS skills, both doctor-staffed and non-doctor-staffed emergency vehicles should be equipped with ultrasound devices, and PMs should be appropriately trained. Pocket ultrasound devices [50,59] would be suitable for this purpose, as the quality of these devices has steadily increased in recent years due to technical innovations [31,32]. The implementation of POCUS by PMs could represent a significant advance for prehospital emergency medicine.

In the long term, a standardized, certified training program for PMs should be developed, establishing POCUS as a permanent fixture in prehospital emergency care. In this context, the integration of specific application examples, such as sonographically assisted identification of the conic ligament, could also be implemented. Furthermore, the use of tele-ultrasound (TELUS) represents another potential development, where remote experts could support PMs in critical situations using POCUS [60,61]. This could significantly improve the availability of specialized sonographic diagnostics in the preclinical setting. Of course, it is important to ensure that the telemedical support never leads to a potentially fatal time delay, such as a delay in securing the patient’s airway.

### 4.4. Limitations

The study was conducted exclusively on healthy volunteers with regular anatomy. This does not account for challenges that arise in real patients, such as anatomical variations, obesity, neck trauma, or limited sonographic windows, which may affect both landmark identification and procedural safety. As such, the transferability of the observed performance to real-world emergency situations with complicating factors remains uncertain (e.g., obesity, tumors) remains to be evaluated [13].

This study did not evaluate the long-term retention of the learned skills [62,63].

The control group did not complete the T_1_-DOPS, so a potential retest bias in the study group cannot be ruled out and may have influenced the observed learning gains. The study group included more male participants than the control group. Although gender-specific effects on ultrasound performance are not well established, a potential influence of gender-related factors such as psychomotor skills cannot be entirely excluded.

## 5. Conclusions

This study demonstrates that paramedics (PMs) can be trained to sonographically identify the conic ligament. After structured courses, PMs were able to reliably identify the conic ligament using point-of-care ultrasound (POCUS), achieving a level of competency comparable to that of EPs. The significant improvement in both objective performance and subjective confidence of the participants in this training underscores the effectiveness of its concept. The findings of this study also highlight the untapped potential of integrating targeted ultrasound education into prehospital emergency care. Broader implementation of POCUS in PM training may enhance the safety and precision of critical airway procedures such as cricothyrotomy. While the results of this study demonstrate that paramedics can be trained to identify the conic ligament using POCUS under controlled conditions, further research is needed to validate the real-world applicability of these skills in emergency settings with complex or altered anatomy. Future research should also focus on long-term skill retention and the development of standardized, certifiable POCUS curricula for non-physician providers.

## Data Availability

The data presented in this study are available on request from the corresponding author. The data are not publicly available because of institutional and national data policy restrictions imposed by the ethics committee since the data contain information that could potentially identify study participants. Data are available upon request (contact via weimer@uni mainz.de) for researchers who meet the criteria for access to confidential data (please provide the manuscript title with your inquiry).

## Supplementary Materials

The following supporting information can be downloaded at: https://www.mdpi.com/article/10.3390/diagnostics15101296/s1, Supplementary Materials S1: pre-survey and post-questionnaire; Supplementary Materials S2: DOPS test for sonographic identification of the conic ligament; Supplementary Materials S3: Baseline characteristics of the study and control group; Supplementary Materials S4: Direct observation of procedural skills (DOPS) test results of sonographic identification and marking of the conic ligament before and after completion of the training program. For the structures to be recognized, see S2; Supplementary Materials S5: Analysis of possible influencing factors on the results of the DOPS test and implementation time for the study group of paramedics; Supplementary Materials S6: Analysis of possible influencing factors on the results of the DOPS test and implementation time for the control group of emergency physicians; Supplementary Materials S7: subjective competence development and comparison of the study group and control group.
