# Supplementary material for: Paramedics Performed Sonographic Identification of the Conic Ligament—A Prospective Controlled Trial
Source: Diagnostics (Basel). 2025 May 21;15(10):1296. doi: 10.3390/diagnostics15101296 (PMC12109798; doi:10.3390/diagnostics15101296)
Supplement: Supplementary file 1 [file diagnostics-15-01296-s001.zip › Supplement 3.pdf]

Supplement 3 Baseline characteristics of the study and control group

Item	N=92 study group	N=28 control group	p- value
**General data**			
Gender n (%)			<0.01
Male	73 (79.4)	12 (42.9)	
Female	14 (15.2)	16 (57.1)	
Divers	0	0	
No answer	5 (5.4)	0	
Age MW ± SD	35.3 ±10.4	36.7±11.7	0.66
**Vorerfahrung**			
work in a preclinical setting n (%)			<0.01
No	14 (15.2)	17 (60.7)	
No answer	8 (8.7)	0	
yes	70 (76.1)	11 (39.3)	
If yes, wow many preclinical cases MW ± SD	4906±4503	660.5±1005.8	<0.01
already taken a course(s) in ultrasound diagnostics n (%)			<0.01
No	78 (84.8)	7 (25)	
No answer	5 (5.4)	0	
yes	9 (9.8)	21 (75)	
If yes, Did these courses teach the sonoanatomy of the larynx/trachea			0.32
yes n (%)	0	4 (14.3)	
no n (%)	9 (9.8)	17 (60.7)	
ultrasound scans (regardless of the region) performed so far MW ± SD	5.0±14.1	554.3±945.7	<0.01
ultrasound scans of the larynx/tracheal region performed so far MW ± SD	0.2±0.9	19.0±52.3	<0.01
Coniotomy seen n (%)			0.36
Yes	48 (52.2)	12 (42.9)	
No	39 (42.4)	16 (57.1)	
No answer	5 (5.4)	0	

coniotomy under sonographic assistance seen<br>n (%)			0.70
Yes	7 (7.6)	1 (3.6)	
No	80 (87)	27 (96.4)	
No answer	5 (5.4)	0	
Coniotomy performed n (%)			0.53
Yes	16 (17.4)	3 (10.7)	
MW±SD	3.6 ±3.9	1.7±0.5	0.42
No	71 (77.2)	25 (89.3)	
No answer	6 (6.5)	0	
Tracheotomy performed n (%)			<0.01
Yes	4 (4.4)	12 (42.9)	
MW±SD	1.5±0.6	8.7±13.5	0.3
No	82 (89.1)	16 (57.1)	
No answer	6 (6.5)	0	
coniotomy under sonographic assistance performed n (%)			0.58
Yes	0	0	
No	86 (93.5)	28 (100)	
No answer	6 (6.5)	0	
Tracheotomy under sonographic assistance performed n (%)			0.40
Yes	2 (2.2)	2 (7.1)	
Anzahl MW	1.5±0.5	1.5±0.5	1.0
No	84 (91.3)	26 (92.9)	
No answer	6 (6.5)	0	
experience of using a “pocket” sonography device n (%)			0.14
No	59 (64.1)	14 (50)	
No answer	6 (6.5)	0	
yes	27 (29.4)	14 (50)	
If yes, number of examinations MW ± SD	13.9±21.1	61.2±89.6	0.01
