# Supplementary material for: Paramedics Performed Sonographic Identification of the Conic Ligament—A Prospective Controlled Trial
Source: Diagnostics (Basel). 2025 May 21;15(10):1296. doi: 10.3390/diagnostics15101296 (PMC12109798; doi:10.3390/diagnostics15101296)
Supplement: Supplementary file 1 [file diagnostics-15-01296-s001.zip › Supplement 4.pdf]

Supplement 4 Direct Observation of Procedural Skills (DOPS) test results of sonographic identification and marking of the conic ligament before and after completion of the training program. For the structures to be recognized see Supplement 2

**Item**	**Points scored study group T1 MW ±SD**	**Points scored study group T2 MW ±SD**	**Delta T1-T2 study group MW ±SD**	**p-value (T1-T2 study group)**	**Points scored control group T2 MW ±SD**	**p-value (study- vs. control group)**
**total points DOPS:** (max. 38 P)	11.2±7.0	31.5±8.4	20.3 ±10.9	<0.001	32.1±5.3	0.81
**%**	29.5±18.4	82.9±22.1	53.4±28.7		84.5±13.9	
**placement and landmarks** (max. 4 P)	2.4±1.2	3.6±0.9	1.2±1.6	<0.001	3.7±0.6	1.0
**%**	60±30	90±22.5	30±40		92.5±15	
placement (max. 2 P)	1.4±0.7	1.9±0.3	0.5±0.8	<0.001	1.7±0.5	0.26
%	70±35	95±15	25±40		85±25	
Landmarks (max. 2 P)	1.0±0.7	1.9±0.4	0.9±0.8	<0.001	1.8±0.5	1.0
%	50±35	95±20	45±40		90±25	
**Transducer handling + image optimization** (max. 8 P)	3.4±2.2	6.9±1.7	3.5±2.8	<0.001	7.2±1.1	0.02
**%**	42.5±27.5	86.3±21.3	43.8±35		90±13.8	
Orientation (max. 2 P)	1.1±0.7	1.8±0.4	0.8±0.8	<0.001	1.9±0.3	1.0
%	55±35	90±20	40±40		95±15	
Positioning (max. 2 P)	0.9±0.7	1.9±0.4	0.9±0.8	<0.001	1.8±0.4	1.0
%	45±35	95±20	45±40		90±20	
Coupling of the transducer (max. 2 P)	0.9±0.7	1.9±0.3	0.9±0.8	<0.001	1.9±0.3	1.0
%	45±35	95±15	45±40		95±15	
image optimization (max. 2 P)	0.5±0.6	1.6±0.5	1.1±0.8	<0.001	2.0±0.2	0.02
%	25±30	80±25	55±40		100±10	

**structure recognition/demonstration** (max. 8 P)	0.8±1.3	6.4±2.2	5.5±2.5	<0.001	6.6±1.7	0.83
**%**	10±16.3	80±27.5	68.8±31.3		82.5±21.3	
structure recognition/demonstration I (max. 3P)	0.4±0.7	2.5±0.9	2.1±1.1	<0.001	2.7±0.7	0.74
%	13.3±23.3	83.3±30	70±36.7		90±23.3	
structure recognition/demonstration II (max. 2P)	0.2±0.5	1.7±0.6	1.5±0.8	<0.001	1.5±0.7	0.13
%	10±25	85±30	75±40		75±35	
structure recognition/demonstration III (max. 3P)	0.2±0.6	2.4±1.1	2.2 ±1.2	<0.001	2.4±0.8	0.53
%	6.7±20	80±36.7	73.3±40		80±26.7	
**Examination procedure** (max. 12 P)	3.4±2.5	9.7±3.1	6.3±3.9	<0.001	9.5±2.2	0.85
**%**	28.3±20.8	80.8±25.8	52.5±32.5		79.2±18.3	
Transverse view trachea (max. 4 P)	1.3±1.2	3.5±0.9	2.2±1.5	<0.001	3.5±0.9	0.92
%	32.5±30	87.5±22.5	55±37.5		87.5±22.5	
sagittal view I (max. 4P)	0.8±1.1	3.5±0.9	2.6±1.4	<0.001	3.2±1.0	0.72
%	20±27.5	87.5±22.5	65±35		80±25	
sagittal view II (max. 4 P)	1.3±0.7	3.2±1.1	1.9±1.3	<0.001	2.8±1.0	0.13
%	32.5±17.5	80±27.5	47.5±32.5		70±25	
**marking the conic ligament (max. 6 P)**	1.2±1.9	5.2±1.8	4.1 ±2.8	<0.001	5.0±1.4	0.87
**%**	20±31.7	86.7±30	68.3±46.7		83.3±23.3	
**Time needed** (max. 180 sec)	158±37	70±34	88±46	<0.001	94±43	<0.01
**%**	87.8±20.6	38.9±18.9	48.9±25.6		52.2±23.9	
