# Supplementary material for: Paramedics Performed Sonographic Identification of the Conic Ligament—A Prospective Controlled Trial
Source: Diagnostics (Basel). 2025 May 21;15(10):1296. doi: 10.3390/diagnostics15101296 (PMC12109798; doi:10.3390/diagnostics15101296)
Supplement: Supplementary file 1 [file diagnostics-15-01296-s001.zip › Supplement 5.pdf]

Supplement 5 Analysis of possible influencing factors on the results of the DOPS test and implementation time for the study group of paramedics

**Item**	**Factor**		**p-value**
**Gender**	**Male**	**Female**	
DOPS T1 (mean ±SD)	11.0±6.9	12.5±7.7	0.57
DOPS T2 (mean ±SD)	32.8±5.3	33.9±4.1	0.51
Time T1 (sec.)	161±38	157±32	0.66
Time T2 (sec.)	72 ±31	77±31	0.54
**current level of training**	**Paramedic with extended training**	**paramedic**	
DOPS T1 (mean ±SD)	11.3±7.2	8.8±4.9	0.465
DOPS T2 (mean ±SD)	32.5±5.2	35.2±2.9	0.272
Time T1 (sec.)	161±38	165±36	0.89
Time T2 (sec.)	75±32	60±25	0.22
**work in a preclinical setting**	**yes**	**no**	
DOPS T1 (mean ±SD)	11.7±7.2	6.4±2.4	**0.009**
DOPS T2 (mean ±SD)	33.1±5.1	31.1±5.7	0.238
Time T1 (sec.)	158±40	178±17	0.083
Time T2 (sec.)	73±32	80±31	0.43
**already taken a course(s) in ultrasound diagnostics**	**yes**	**no**	
DOPS T1 (mean ±SD)	13.2±8.2	11.0±6.9	0.37
DOPS T2 (mean ±SD)	33.4±8.4	32.9±4.7	0.17
Time T1 (sec.)	157±32	161±38	0.68
Time T2 (sec.)	70±29	73±32	0.91
**ultrasound examination independently**	**yes**	**no**	
DOPS T1 (mean ±SD)	12.6±8.4	10.7±6.3	0.46
DOPS T2 (mean ±SD)	33.6±5.8	32.6±4.6	0.14
Time T1 (sec.)	156±35	162±37	0.38
Time T2 (sec.)	68±31	75±31	0.24
**Coniotomy seen**	**yes**	**no**	
DOPS T1 (mean ±SD)	10.6±6.2	12.1±8.0	0.5
DOPS T2 (mean ±SD)	32.1±5.7	34.1±4.0	0.12
Time T1 (sec.)	161±39	159±34	0.96
Time T2 (sec.)	76 ±35	69±26	0.67
**coniotomy under sonographic assistance seen**	**yes**	**no**	
DOPS T1 (mean ±SD)	12.7±5.8	11.1±7.1	0.41
DOPS T2 (mean ±SD)	31.7±5.4	33.1±5.1	0.44
Time T1 (sec.)	144±33	162±37	0.16
Time T2 (sec.)	71±26	73±32	0.91
**Coniotomy performed**	**yes**	**no**	

DOPS T1 (mean ±SD)	11.2±5.9	11.3±7.3	0.77
DOPS T2 (mean ±SD)	31.9±7.1	33.1±4.6	0.92
Time T1 (sec.)	169±42	159±36	0.57
Time T2 (sec.)	67±31	73±31	0.42
**experience of using a “pocket” sonography device**	**yes**	**no**	
DOPS T1 (mean ±SD)	11.4±7.2	11.3±7.2	0.88
DOPS T2 (mean ±SD)	34.1±5.4	32.4±5.0	0.05
Time T1 (sec.)	158±36	162±37	0.52
Time T2 (sec.)	66±30	76±32	0.12
