# Supplementary material for: Paramedics Performed Sonographic Identification of the Conic Ligament—A Prospective Controlled Trial
Source: Diagnostics (Basel). 2025 May 21;15(10):1296. doi: 10.3390/diagnostics15101296 (PMC12109798; doi:10.3390/diagnostics15101296)
Supplement: Supplementary file 1 [file diagnostics-15-01296-s001.zip › Supplement 6.pdf]

Supplement 6 Analysis of possible influencing factors on the results of the DOPS test and implementation time for the control group of emergency physicians

Item	Factors		p-value
**Gender**	**Male**	**Female**	
DOPS T2 (mean±SD)	34.9±3.4	30.6 ±5.8	0.1
Time T2 (sec.)	96.6±37.2	93.2±48.3	1.0
**borad certification in emergency medicine**	**yes**	**no**	
DOPS T2 (mean±SD)	32.9±4.8	30.8±6.4	0.46
Time T2 (sec.)	92.6±44.2	96,9±46.3	0.7
**work in a preclinical setting**	**yes**	**no**	
DOPS T2 (mean±SD)	34.0 ±3.7	30.6±6.2	0.21
Time T2 (sec.)	92.6±34.5	95.6±51.2	0.50
**Coniotomy seen**	**yes**	**no**	
DOPS T2 (mean±SD)	32.1±5.6	32.1±5.6	0.88
Time T2 (sec.)	94.9±51.8	94.0±39.8	0.98
**Tracheotomy performed**	**yes**	**no**	
DOPS T2 (mean±SD)	31.1±4.9	32.7±5.8	0.37
Time T2 (sec.)	95.2±42.6	93.9±46.5	0.98
**experience of using a "pocket" sonography device**	**yes**	**no**	
DOPS T2 (mean±SD)	32.2±4.9	32.0±6.0	0.83
Time T2 (sec.)	91.4±46.4	96.5±44.1	0.7
