# Supplementary material for: Paramedics Performed Sonographic Identification of the Conic Ligament—A Prospective Controlled Trial
Source: Diagnostics (Basel). 2025 May 21;15(10):1296. doi: 10.3390/diagnostics15101296 (PMC12109798; doi:10.3390/diagnostics15101296)
Supplement: Supplementary file 1 [file diagnostics-15-01296-s001.zip › Supplement 7.pdf]

Supplement 7 subjective competence development and comparison of the study group and control group

**competence area (1= very good; 7=low)**	**T1 study group MW ±SD N=92**	**T1 control group MW ±SD N=28**	**P-value (study-vs. control group T1)**	**T2 study group MW ±SD N=92**	**T2 control group MW ±SD N=28**	**P-value (study-vs. control group T2)**	**Delta T1-T2 study group MW ±SD (p-value) N=92**	**Delta T1-T2 control group MW ±SD (p-value) N=28**	**P-Wert Delta-comparison (study-vs. control group)**
**Overall score competence assessment**	4.8±0.9	3.5±1.1	<0.01	2.5±0.9	2.0±0.6	<0.01	1.9±1.0 (<0.001)	1.1±1.0 (<0.001)	<0.01
**overall score manual examination**	4.4±1.6	3.3±1.3	0.01	2.5 ±1.0	2.1±0.9	0.03	1.6±1.5 (<0.001)	1.1±0.9 (<0.001)	<0.01
Manual examination of the neck	4.7±1.7	3.3±1.3	<0.01	2.8±1.2	2.2±1.0	0.06	1.9±1.8 (<0.001)	0.9±0.8 (<0.001)	<0.01
Manual examination of the larynx	4.1±1.8	3.5±1.4	0.42	2.3±1.0	1.9±1.0	0.10	1.8±1.6 (<0.001)	1.5±1.2 (<0.001)	0.67
**overall score anatomical knowledge**	3.6±1.3	3.2±1.2	0.34	2.6±0.9	2.2±0.9	0.05	0.8±1.1 (<0.001)	0.9±1.0 (<0.001)	0.76
Anatomical knowledge of the neck	3.6±1.2	3.1±1.1	0.2	2.7±1.1	2.1±0.8	0.02	0.8±1.2 (<0.001)	1.0±0.9 (<0.001)	0.64
Anatomical knowledge of the larynx/trachea	3.6±1.5	3.3±1.3	0.37	2.5±0.9	2.3±0.9	0.26	1.0±1.4 (<0.001)	1.0±1.1 (<0.001)	0.89
**overall score Sono- Anatomical knowledge of the head-neck**	6.1±1.3	4.3±1.5	<0.01	3.1±1.3	2.2±0.9	<0.01	3.0±1.5 (<0.001)	2.4 ±1.7 (<0.001)	0.009
**Overall score basic ultrasound competencies**	6.2±1.1	2.6±1.3	<0.01	2.5±0.9	1.8±0.7	<0.01	3.2±1.4 (<0.001)	0.7±1.3 (<0.001)	<0.01
Physical basics	4.2±1.6	2.9±1.6	<0.01	2.8±1.2	1.9±0.8	<0.01	1.4±1.8 (<0.001)	1.3±1.9 (<0.001)	1.0
Understanding how ultrasound images are created	3.9±1.6	2.5±1.3	<0.01	2.5±1.1	1.7±0.8	<0.01	1.3±1.5 (<0.001)	0.9±1.4 (<0.001)	0.28
Spatial orientation in ultrasound images	4.2±1.5	2.4±1.2	<0.01	2.4±1.1	1.0±0.7	<0.01	1.8±1.6 (<0.001)	1.1 ±1.4 (<0.001)	0.07
Transducer handling for the examination of the neck	5.6±1.6	3.2±1.7	<0.01	2.5±1.1	2.2±2.0	0.05	3.1±1.8 (<0.001)	1.1±2.4 (<0.001)	<0.01
Image optimization	4.8±1.5	2.7±1.3	<0.01	2.6±1.1	2.0±0.8	<0.01	2.2±1.7 (<0.001)	0.8±1.1 (<0.001)	<0.01
Artifacts (origin and detection)	5.0±1.5	2.8±1.4	<0.01	2.8±1.2	1.8±0.6	<0.01	2.1±1.6	1.1±1.3	0.02

							(<0.001)	(<0.001)	
Patient guidance during the examination	4.9±1.6	2.7±1.6	<0.01	2.2±1.0	1.8±0.8	0.15	2.7±1.8 (<0.001)	1.1±1.5 (<0.001)	<0.01
**score competencies in sonography oft he laryngeal region**	6.2±1.1	5.1±1.5	<0.01	2.3±1.1	1.9±0.6	0.37	3.6±1.5 (<0.001)	2.9±1.8 (<0.001)	0.09
Structuring the standard sections for the examination of the laryngeal region in the sagittal plane	6.1±1.2	4.8±1.4	<0.01	2.3±1.1	2.0±0.7	0.94	3.8±1.6 (<0.001)	3.1±1.6 (<0.001)	0.06
Structuring the standard sections for the examination of the laryngeal region in the transversal plane	6.2±1.2	5.0±1.4	<0.01	2.3±1.1	1.9±0.6	0.62	3.9±1.6 (<0.001)	3.4±1.7 (<0.001)	0.17
Sonographic detection/identification of the conic ligament (emergency coniotomy access)	6.4±1.1	5.0±1.6	<0.01	2.4±1.1	1.9±0.7	0.09	4.0±1.5 (<0.001)	3.3±1.7 (<0.001)	0.22
**Would you be confident to perform such an intervention (emergency coniotomy) with telemedical suppor only?**	4.2±2.2	3.5±2.2	0.42	2.4±1.5	2.0±1.5	0.37	1.8±1.9 (<0.001)	1.4±1.4 (<0.001)	0.79
