# Supplementary material for: Paramedics Performed Sonographic Identification of the Conic Ligament—A Prospective Controlled Trial
Source: Diagnostics (Basel). 2025 May 21;15(10):1296. doi: 10.3390/diagnostics15101296 (PMC12109798; doi:10.3390/diagnostics15101296)
Supplement: Supplementary file 1 [file diagnostics-15-01296-s001.zip › Supplement_1.pdf]

Supplement 1 pre-survey and post-questionnaire

Pre

1.0 general Data

1.1 Code: ____ (please state correctly)

1.2 gender: ☐ male ☐ female ☐ diverse
1.3 age (in years): ________________
1.3 current level of training: ☐ paramedic ☐ paramedic with extended training ☐ medical doctor, resident
☐ medical doctor, specialist ☐ medical doctor, attending

1.4 In which specialty do you work? : ____________________________________

1.5 If applicable, which year of training are you in? __________________________

1.6 Are you board certified in emergency medicine: ☐ yes ☐ no

1.7 Do you work in a preclinical setting: ☐ yes ☐ no

1.8 How many preclinical cases have you handled so far? _________________________

2.0 Prior experience

2. 1 Have you already taken a course(s) in ultrasound diagnostics? ☐ No ☐ yes, the following:

____

2.2 Did these courses teach the sonoanatomy of the larynx/trachea? ☐ No ☐ yes

2.3 How many ultrasound scans (regardless of the region) have you performed so far yourself (with or without guidance) on a real patient? :_____________________ examinations

2.4 How many ultrasound scans of the larynx/tracheal region have you performed so far yourself (with or without guidance) on a real patient ? :_____________________ examinations

2.5 Have you performed or seen a coniotomy?

☐ no ☐ yes

2.6 Have you performed or seen a a coniotomy under sonographic assistance?

☐ no ☐ yes

2.7 How many coniotomies have you performed yourself (with or without guidance) on a real patient?
_____________________ coniotomies

2.8 How many tracheotomies have you performed yourself (with or without guidance) on a real patient?
_____________________ tracheotomies

2.9 How many coniotomies under sonographic assistance have you performed yourself (with or without guidance) on a real patient? _____________________ coniotomies

2.10 How many tracheotomies under sonographic assistance have you performed yourself (with or without guidance) on a real patient? _____________________ tracheotomies

2.11 Do you already have experience of using a “pocket” sonography device?

☐ no ☐ Yes, the following number of examinations: _______________

2.12 How would you rate your performance in the following areas?

	very good/full and complete						Low/ not at all
Manual examination of the neck	☐ 1	☐ 2	☐ 3	☐ 4	☐ 5	☐ 6	☐ 7
Manual examination of the larynx	☐ 1	☐ 2	☐ 3	☐ 4	☐ 5	☐ 6	☐ 7
Anatomical knowledge of the neck	☐ 1	☐ 2	☐ 3	☐ 4	☐ 5	☐ 6	☐ 7
Anatomical knowledge of the larynx/trachea	☐ 1	☐ 2	☐ 3	☐ 4	☐ 5	☐ 6	☐ 7
Sono- Anatomical knowledge of the neck	☐ 1	☐ 2	☐ 3	☐ 4	☐ 5	☐ 6	☐ 7
Sono- Anatomical knowledge of the larynx/trachea							
Physical basics	☐ 1	☐ 2	☐ 3	☐ 4	☐ 5	☐ 6	☐ 7
Understanding how ultrasound images are created	☐ 1	☐ 2	☐ 3	☐ 4	☐ 5	☐ 6	☐ 7
Spatial orientation in ultrasound images	☐ 1	☐ 2	☐ 3	☐ 4	☐ 5	☐ 6	☐ 7
Transducer handling for the examination of the neck	☐ 1	☐ 2	☐ 3	☐ 4	☐ 5	☐ 6	☐ 7
Image optimization	☐ 1	☐ 2	☐ 3	☐ 4	☐ 5	☐ 6	☐ 7
Artifacts (origin and detection)	☐ 1	☐ 2	☐ 3	☐ 4	☐ 5	☐ 6	☐ 7
Patient guidance during the examination	☐ 1	☐ 2	☐ 3	☐ 4	☐ 5	☐ 6	☐ 7
Structuring the standard sections for the examination of the laryngeal region in the sagittal plane	☐ 1	☐ 2	☐ 3	☐ 4	☐ 5	☐ 6	☐ 7
Structuring the standard sections for the examination of the laryngeal region in the transversal plane	☐ 1	☐ 2	☐ 3	☐ 4	☐ 5	☐ 6	☐ 7
Sonographic detection/identification of the conic ligament (emergency coniotomy access)	☐ 1	☐ 2	☐ 3	☐ 4	☐ 5	☐ 6	☐ 7
Would you be confident to perform such an intervention (emergency coniotomy) with telemedical suppor only?	☐ 1	☐ 2	☐ 3	☐ 4	☐ 5	☐ 6	☐ 7
Would you be confident to perform such an intervention (emergency coniotomy) on your own?	☐ 1	☐ 2	☐ 3	☐ 4	☐ 5	☐ 6	☐ 7

Post

1. Code ____ (please state correctly)

2. Did you find the instructional video/lecture helpful?

full and complete **not at all**

☐ ☐ ☐ ☐ ☐ ☐ ☐

3. Did you find the training helpful?

full and complete **not at all**

☐ ☐ ☐ ☐ ☐ ☐ ☐

4. how confident did you feel using the sonographic technique to locate the conic ligament?

complete confidence **not confidet at all**

☐ ☐ ☐ ☐ ☐ ☐ ☐

Individual Feedback

5. How would you rate your performance in the following areas?

	**very good/full and complete**						**Low/ not at all**
Manual examination of the neck	☐ 1	☐ 2	☐ 3	☐ 4	☐ 5	☐ 6	☐ 7

Manual examination of the larynx	☐ 1	☐ 2	☐ 3	☐ 4	☐ 5	☐ 6	☐ 7
Anatomical knowledge of the neck	☐ 1	☐ 2	☐ 3	☐ 4	☐ 5	☐ 6	☐ 7
Anatomical knowledge of the larynx/trachea	☐ 1	☐ 2	☐ 3	☐ 4	☐ 5	☐ 6	☐ 7
Sono- Anatomical knowledge of the neck	☐ 1	☐ 2	☐ 3	☐ 4	☐ 5	☐ 6	☐ 7
Sono- Anatomical knowledge of the larynx/trachea							
Physical basics	☐ 1	☐ 2	☐ 3	☐ 4	☐ 5	☐ 6	☐ 7
Understanding how ultrasound images are created	☐ 1	☐ 2	☐ 3	☐ 4	☐ 5	☐ 6	☐ 7
Spatial orientation in ultrasound images	☐ 1	☐ 2	☐ 3	☐ 4	☐ 5	☐ 6	☐ 7
Transducer handling for the examination of the neck	☐ 1	☐ 2	☐ 3	☐ 4	☐ 5	☐ 6	☐ 7
Image optimization	☐ 1	☐ 2	☐ 3	☐ 4	☐ 5	☐ 6	☐ 7
Artifacts (origin and detection)	☐ 1	☐ 2	☐ 3	☐ 4	☐ 5	☐ 6	☐ 7
Patient guidance during the examination	☐ 1	☐ 2	☐ 3	☐ 4	☐ 5	☐ 6	☐ 7
Structuring the standard sections for the examination of the laryngeal region in the sagittal plane	☐ 1	☐ 2	☐ 3	☐ 4	☐ 5	☐ 6	☐ 7
Structuring the standard sections for the examination of the laryngeal region in the transversal plane	☐ 1	☐ 2	☐ 3	☐ 4	☐ 5	☐ 6	☐ 7
Sonographic detection/identification of the conic ligament (emergency coniotomy access)	☐ 1	☐ 2	☐ 3	☐ 4	☐ 5	☐ 6	☐ 7
Would you be confident to perform such an intervention (emergency coniotomy) with telemedical suppor only?	☐ 1	☐ 2	☐ 3	☐ 4	☐ 5	☐ 6	☐ 7
Would you be confident to perform such an intervention (emergency coniotomy) on your own?	☐ 1	☐ 2	☐ 3	☐ 4	☐ 5	☐ 6	☐ 7
