# Supplementary material for: Paramedics Performed Sonographic Identification of the Conic Ligament—A Prospective Controlled Trial
Source: Diagnostics (Basel). 2025 May 21;15(10):1296. doi: 10.3390/diagnostics15101296 (PMC12109798; doi:10.3390/diagnostics15101296)
Supplement: Supplementary file 1 [file diagnostics-15-01296-s001.zip › Supplement_2.pdf]

Supplement 2 DOPS test for songorpah identification oft he Conic Ligament

Participant code: **Auditor:**
Time of audit: ☐ before training ☐ after training

**☐ case 1 vignette (for pocket device)::**
You are an emergency physician/paramedic and are providing pre-hospital care to a patient with a vital airway obstruction. Intubation and the use of supraglottic airway aids have so far been unsuccessful. As the last option in the "cannot-ventilate-cannot-intubate" (CICV) situation, you plan to perform an emergency coniotomy. You would first like to sonographically locate the conic ligament and mark it with a cannula.
**Task 1**: Place the patient in an appropriate position and show the **landmarks** jugulum and cricoid cartilage with your finger. **(4 points)**

Proper positioning (head hyperextended, positioning aid under shoulders if necessary)	2 ☐		Both landmarks shown correctly	2 ☐
Positioning only carried out correctly when supported verbally	1 ☐		Only one landmark shown correctly	1 ☐
Positioning only carried out correctly when supported manually	0 ☐		No landmark shown correctly	0 ☐

Task 2: First locate the **transverse view of the trachea** as an orientation plane. Starting from this, show the tracheal cartilage, cricoid cartilage and thyroid cartilage in **several sagittal views** and identify the area of the conic ligament. **Show the structures**: ***thyroid gland, trachea/tracheal cartilage, cricoid cartilage, thyroid cartilage, infrahyoid muscles and the conic ligament***

Transducer handling (max. 8 Points)

Orientation			Positioning			Coupling of the transducer			image optimization	
Correct, or immediately checked by the user based on the image movement/by decoupling	2 ☐		Correct or immediately transferred from another view	2 ☐		Transducer is well attached with sufficient amount of gel, constant pressure	2 ☐		Independent and adequate adjustment of appropriate image parameters (gain, penetration depth, focus) even during examination	2 ☐
Adjusts after initial difficulties/ after verbal help	1 ☐		Adjusts after initial difficulties/ after verbal help	1 ☐		Adjusts after initial difficulties/ after verbal help	1 ☐		Adjusts after initial difficulties/ after verbal help	1 ☐
Correct orientation only with manual help	0 ☐		Correct positioning only with manual help	0 ☐		No sufficient pressure and/or no gel + unregulated pressure / transducer partly in contact with air	0 ☐		No adequate continuous image optimization despite request, manual help necessary	0 ☐

© J. Weimer et. al.

Task 3 Examination procedure (12 points) + structure recognition/demonstration (8 points)

**Transverse view** trachea correctly adjusted; trachea centrally in the image, tracheal cartilages and air reflexes can be seen, adequate speed	4 ☐
Transverse view trachea adequately adjusted; sometimes verbal assistance necessary or inadequate speed	2 ☐
Transverse view trachea only adjusted with manual help	0 ☐

Reference image: correctly named structures ☐ **thyroid gland** ☐ **trachea** ☐ **infrahyoid muscles**

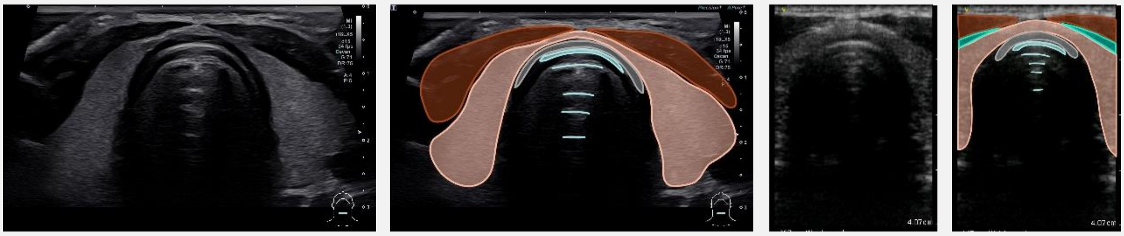

Correct transfer to **sagittal view I**: tracheal cartilages ("pearl necklace") visible, T1 cartilage shown as an elongated structure, parts of the cricoid cartilage visible, adequate speed	4 ☐
Inadequate transfer to sagittal view I, not all structures visible, sometimes verbal help necessary or inadequate speed	2 ☐
sagittal view I only adjusted with manual help	0☐

Reference image: correctly named structures ☐**tracheal cartilage** ☐ **cricoid cartilage**

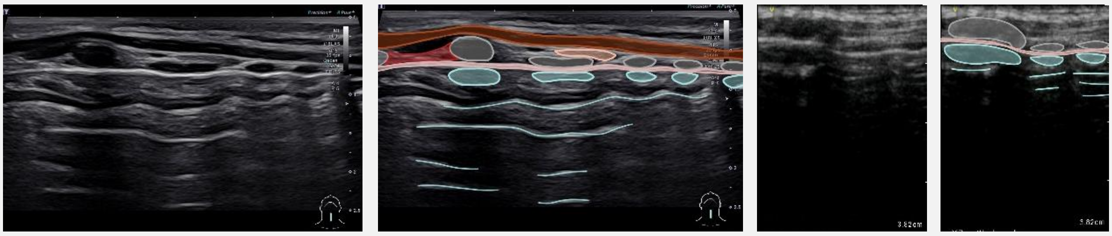

Correct transfer to **sagittal view II:** cricoid cartilage and thyroid cartilage visible, adequate speed	4 ☐
Inadequate transfer to sagittal view II, not all structures visible, sometimes verbal help necessary or inadequate speed	2 ☐
sagittal view II only adjusted with manual help	1 ☐

Reference image: correctly named structures ☐ **cricoid cartilage** ☐ **thyroid cartilage** ☐ **conic ligament**

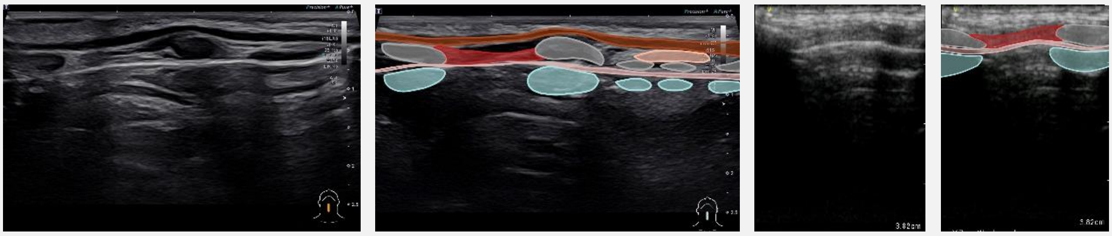

Task 4: With the aid of a cannula, **mark the spot** (acoustic shadow) where the conic ligament is located (6 points)

correct marking	6 ☐
correct marking only with verbal help	3 ☐
correct marking only with manual help	0 ☐

Reference images

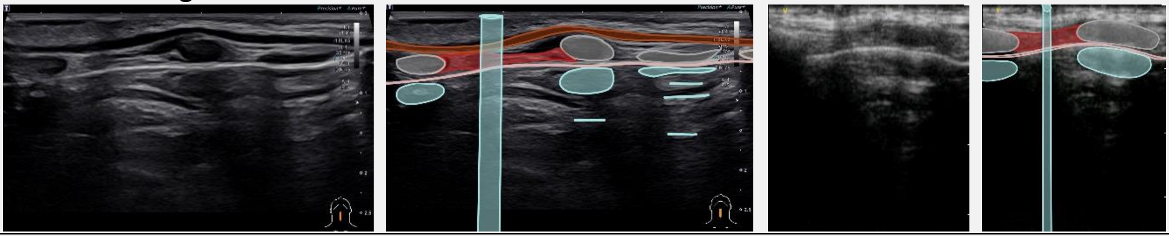

Time:

total points:

© J. Weimer et. al.
